# Epidemiology of Occult Hepatitis B Infection Among Thalassemic, Hemophilia, and Hemodialysis Patients

**DOI:** 10.5812/hepatmon.5934

**Published:** 2012-05-30

**Authors:** Mohammad Kazemi Arababadi, Behzad Nasiri Ahmadabadi, Hassan Yousefi Daredor, Derek Kennedy

**Affiliations:** 1 Department of Microbiology, Hematology and Immunology, Rafsanjan University of Medical Sciences, Rafsanjan, IR Iran; 2 Immunology of Infectious Diseases Research Center, Rafsanjan University of Medical Sciences, Rafsanjan, IR Iran; 3 Department of Biochemistry, Rafsanjan University of Medical Sciences, Rafsanjan, IR Iran; 4 Schools of Biomolecular and Physical Science, Griffith University Nathan, Queensland, Australia

**Keywords:** Blood Donors, Thalassemia, Hemophilia

## Abstract

**Context:**

Hepatitis B virus (HBV) is the most common disease commuted through blood transfusion. Occult hepatitis B infection (OBI) is a form of the disease which does not present Hepatitis B surface antigens (HBsAg) in the serum of patients; however, HBV-DNA is detectable in the serum and hepatocytes of patients. OBI is an important risk factor to induce post transfusion hepatitis (PTH), cirrhosis, hepatocellular carcinoma (HCC) and reactivation of the HBV. Recently, several reports from various regions of the world have been published regarding PTH among blood recipients as well as HCC, and cirrhosis among patients who require permanent blood transfusion, including diseases such as hemophilia, hemodialysis and thalassemia. This form of the hepatitis also creates problems for individuals that are co-infected with other viruses such as HCV and HIV. To determine the prevalence of OBI among hemophilia, hemodialysis and thalassemia patients is important because it is a high risk factor for PTH, HCC and cirrhosis therefore, its detection is a critical strategy for most health care services. This review addresses recent information regarding prevalence of OBI in relation to the mentioned diseases.

**Evidence Acquisition:**

The data presented here was collected by searching the key words in Pubmed and Scopous databases.

**Results:**

Our searching in the published papers revealed that OBI prevalence is frequent in patients receiving frequent blood transfusions.

**Conclusions:**

it seems that one of the main mechanisms for OBI transmission is most likely through infected blood and its component and evaluation of the prevalence of OBI in donors and patients, especially those with hemophilia and thalassemia should be foul considered.

## 1. Context

Hepatitis B virus (HBV) is the most prevalent cause of human liver disease [1][2]. Clinical presentation of the hepatitis B varies among individuals but the most frequent symptom is the alteration of scleroproteins and nausea caused by the increase of total bilirubin levels and malfunction of the liver cells, respectively [3][4]. Investigations have reported that a lot of individuals suffer from the long term forms of HBV infection including chronic, asymptomatic and occult (OBI) hepatitis B infection [5][6]. To avoid transmission, most blood transfusion services are using ELISA techniques to detect HBsAg, and to decrease the possibility of HBV infection, but this is not 100% effective and cases of post transfusion hepatitis (PTH) have been reported [7]. OBI is a clinical form of hepatitis B in which, despite the absence of detectable hepatitis B surface antigens (HBsAg) in serum, HBV-DNA is present in both serum and hepatocytes [8]. Therefore, OBI seems to be the most likely mechanism for PTH in permanent blood recipients including, hemophilia, thalassemia patients and those requiring hemodialysis (HD) [6][9]. It is important to examine the prevalence of OBI in blood products so that we may gain insights and suitable diagnosis plans for transfusion services, considering the impact that OBI has when transmitted via blood, and its components, to recipients,. Previous studies have revealed that OBI can be related to several pathologic features of liver including hepatocellular carcinoma, ﬁbrosis and cirrhosis [1][10]. Therefore, OBI can be considered as a potential risk factor for patients such as those with thalassemia, hemophilia and potentially all other patients requiring hemodialysis. Additionally, due to the fact that diseases such as HCV, HBV and HIV, which are transmitted through blood transfusion, are readily manifested in thalassemic, HD and hemophilia patients, there is an urgent need to address blood contamination with a view to curb disease transmission through transfusion. Hence, in this article, we have collated several reports regarding OBI prevalence in hemophilia, thalassemic and HD patients.

## 2. Evidence Acquisition

The data presented here was collected by searching the following key words in Pubmed and Scopous databases: Occult HBV infection, Blood donors, Thalassemia, Hemophilia, Hemodialysis and Co-infection. All the papers regarding the prevalence of OBI among thalassemic, hemodialysis and hemophilia patients were used and their data were used in the current review article.

### 2.1. Status of OBI Prevalence Among Hemophilia Patients

Hemophilia patients receive a large volume of blood and its components, especially coagulant factors VIII and IX, hence, they are at extreme risk of acquiring infectious diseases through blood transfusion. However, globally there are few reports regarding OBI prevalence among patients with hemophilia. Toyoda et al., reported that OBI prevalence among Japanese hemophilia patients was 51.2 % [11] (Table 1). Borhany and colleagues reported that the rate of OBI prevalence among hemophilia patients in Pakistan was 1.73 % [12]. However, another study by Windyga et al. on 115 patients with hemophilia demonstrated that none of the evaluated patients had been infected with OBI [13]. There are signiﬁcant differences in the reported rates of OBI in these patients and it is to be determined what factors contribute to the variability. One of the possible causes of the discrepancy is that developed countries, such as Japan, have more rigorous surveillance mechanisms in place through their health services, thus, are able to detect HBV-DNA more comprehensively. Therefore, they are in a position to report the high range of OBI among hemophilia patients more accurately.

**Table 1:** The Prevalence of OBI and HBV Serological Markers Among Blood Recipient Patients

	**Disease**	**Country**	**Region, City**	**Prevalence of OBI a, %**	**Prevalence, %**		
					**Anti HBc**	**Anti HBs**	**HBs Ag**
Toyoda et al. [11] (2004)	Hemophilia	Japan	ND	51.2	86	62.8	0
Borhany et al. [12] (2011)	Hemophilia	Pakistan	Karachi	1.73	ND a	ND	ND
Windyga et al. [13] (2006)	Hemophilia	Polish	ND	0	69.5	49.5	7.8
Aghakhani et al. [18] (2010)	Hemodialysis	Iran	Tehran	3.11	6.2	77.5	2.8
Arababadi et al. [2] (2009)	Hemodialysis	Iran	Kerman	0	33.3	40.7	0
Di Stefano et al. [20] (2009)	Hemodialysis	Italia	ND	26.6	72	31	ND
Abu-El-Makarem et al. [22] (2012)	Hemodialysis	Egypt	ND	4.13	20	ND	ND
Sav et al. [25] (2010)	Hemodialysis	Turkey	ND	16.9	ND	ND	ND
Kanbay et al. [24] (2006)	Hemodialysis	Turkey	ND	15.2	ND	ND	ND
Goral et al. [28] (2006)	Hemodialysis	Turkey	ND	0	ND	ND	0
Siagris et al. and Mina et al. [10][29] (2010)	Hemodialysis	Greece	ND	0.9-20.4	47.8	66.9	5.5
Motta et al. [31] (2010)	Hemodialysis	Brasilia	ND	15	3	27	0
Gabrerizo et al. [30] (1997)	Hemodialysis	Italy	ND	58	ND	ND	ND
Gwak et al. [32] (2008)	Hemodialysis	South-Korea	ND	0	67.4	69.8	4.8
Jain et al. [23] (2008)	Hemodialysis	China	ND	5	5	ND	11
Singh et al. [17] (2003)	Thalassemia	India	ND	31.4	20	75.7	5.7
Arababadi et al. [14] (2008)	Thalassemia	Iran	Kerman	0	33	40.7	0

^a^ Abbreviations: ND, not determinant; OBI, occult hepatitis B infection

Since OBI can lead to several diseases such as cirrhosis, and hepatocellular carcinoma (HCC), more studies regarding the prevalence of OBI in the hemophilia patients is urgently needed so that we can ascertain the risk to recipients. Especially if there are treatments or screening regimes in place which can directly reduce the passage of HBV through blood transfusion. However, the high variance between incidences of OBI in the current publications (51.2 % in Japan [11] and 0 % in Poland [13]) encouraged us to evaluate an additional parameter that may inﬂuence transmission of the disease and that is the epidemiology of OBI in thalassemic patients.

### 2.2. Status of OBI Prevalence Among Thalassemic Patients

Thalassemia patients, like those with hemophilia are “at risks” because they receive high volumes of transfused blood and its components [14]. Although, there are several publications regarding HBV infection prevalence in thalassemic patients [15][16], there are only two papers regarding the prevalence of the OBI form of HBV infection among the patients. Singh et al. from India reported that OBI had a high prevalence among thalassemic patients with a prevalence of 31.4 % [17] (Table 1). In contrast with Singh et al., our previous study on the 60 patients showed that there were no OBI cases among Iranian thalassemic patients [14]. It seems that more information is needed in this ﬁeld to design a suitable plan for diagnosis and treatment of OBI in the thalassemic patients. Like the hemophilia patients, it seems that the sample size of the studies and also the power and ﬁdelity of the technique to detect HBV-DNA can inﬂuence the accuracy of the results of the mentioned studies. Therefore, the variable reported between India and Iran, which arelocated in the same region, may not reﬂect what is happening in the community and larger sample sizes using more powerful techniques are required.

### 2.3. Status of OBI Prevalence Among Hemodialysis Patients

In contrast to thalassemia and hemophilia, signiﬁcant data are available regarding the epidemiology of the OBI in hemodialysis (HD) patients. For instance, our previous study showed that none of the HCV infected HD patients were infected with OBI [2]. Contrary to our results, Aghakhani et al., reported that 3.11 % (Table 1) of Iranian HD patients with isolated anti-HBc were HBsAg-/ HBV-DNA+[18]. Studies on Italian HD patients revealed that 26.6 % of them were OBI positive [19][20]. American investigators demonstrated that 9 (3.8 %) out of 239 HD patients suffered from OBI [21]. Abu and colleagues evaluated 145 HD patients from Egypt and they reported that 4.13 % of their patients suffered from OBI [22]. Signiﬁcantly high occurrences of OBI were reported by Sav et al. [23] and Kanbay et al. [24] from Turkey. Their study showed that 16.9 % and 15.2 % of the patients on HD were infected with OBI, respectively [25]. Interestingly, another study from Turkey revealed that OBI prevalence among HD patients was 17-27.5 % [26][27]. In apparent contradiction with the mentioned Turkish study, Goral et al., revealed that the rate of OBI prevalence among Turkish HD patients was zero [28], it is difficult to reconcile these varying statistics. In contrast with the Turkish researchers, studies from Greece showed that 0.9-20.4 % of the HD patients suffered from OBI [10][29]. Interestingly, the highest OBI prevalence among HD patients was reported by Cabrerizo et al., (58%) from Spain in 1997 [30].OBI prevalence among Brazilian HD patients was reported by Motta and colleagues [31]. They showed the occurrence to be 15 % among the patients [31]. Investigators from South-Korea [32] and China [23] revealed that OBI prevalence among patients on HD were 0 and 5 %, respectively.

## 3. Results

Based on the reports listed above it seems that the prevalence of OBI among HD patients varies and is not regional speciﬁc because high OBI prevalence was seen across a broad range of countries with no obvious pattern suggesting the cause of the variability.

## 4. Conclusions

According to the reports mentioned above, it can be concluded that the prevalence of OBI is frequent in patients receiving frequent blood transfusions. Moreover, the main mechanism for HBV infection in patients is through blood and its components, hence, it can be concluded that more sensitive screening tests, such as PCR, should be employed to decrease the risk of post transfusion hepatitis. In conclusion, it seems that OBI has a high prevalence in the mentioned blood recipient patients and one of the main mechanisms for this transmission is most likely through infected blood and its components. Therefore, it is proposed that the directors of health services should accurately evaluate the prevalence of OBI in donors and patients, especially those with hemophilia and thalassemia because of the limited data available. OBI can be considered as a risk factor for hepatocellular carcinoma, ﬁbrosis and cirrhosis [1][10], hence, screening the thalassemic, HD and also hemophilia patients with respect to OBI is strongly recommended. In addition, based on the data indicating that HCV co-infection can be considerd as a main cause in the development of OBI, evaluation of HCV infection in permanent blood recipients is recommended by the authors. Finally, the sample size, power, ﬁdelity and sensitivity of PCR screening, as well as sources of the population can affect the results of the studies. The evaluated researches presented in the current review are not matched regarding the above variables due to the different requirements of blood services or research groups in the countries canvassed for this review; hence, there is an urgent need to deﬁne a single universal screening protocol that can be applied to all nations so that data can be accurately and reliably reported around the world, only then can we hope to launch a uniﬁed campaign designed to constrain the spread of these diseases.
